# Metagenomic evidence for metabolism of trace atmospheric gases by high-elevation desert Actinobacteria

**DOI:** 10.3389/fmicb.2014.00698

**Published:** 2014-12-17

**Authors:** Ryan C. Lynch, John L. Darcy, Nolan C. Kane, Diana R. Nemergut, Steve K. Schmidt

**Affiliations:** ^1^Department of Ecology and Evolutionary Biology, University of ColoradoBoulder, CO, USA; ^2^Environmental Studies Program, University of ColoradoBoulder, CO, USA; ^3^Institute of Arctic and Alpine Research, University of ColoradoBoulder, CO, USA; ^4^Department of Biology, Duke UniversityDurham, NC, USA

**Keywords:** aerobiology, Atacama Desert, methylotrophy, *Pseudonocardia*, trace gas oxidation

## Abstract

Previous surveys of very dry Atacama Desert mineral soils have consistently revealed sparse communities of non-photosynthetic microbes. The functional nature of these microorganisms remains debatable given the harshness of the environment and low levels of biomass and diversity. The aim of this study was to gain an understanding of the phylogenetic community structure and metabolic potential of a low-diversity mineral soil metagenome that was collected from a high-elevation Atacama Desert volcano debris field. We pooled DNA extractions from over 15 g of volcanic material, and using whole genome shotgun sequencing, observed only 75–78 total 16S rRNA gene OTUs_3%_. The phylogenetic structure of this community is significantly under dispersed, with actinobacterial lineages making up 97.9–98.6% of the 16S rRNA genes, suggesting a high degree of environmental selection. Due to this low diversity and uneven community composition, we assembled and analyzed the metabolic pathways of the most abundant genome, a *Pseudonocardia* sp. (56–72% of total 16S genes). Our assembly and binning efforts yielded almost 4.9 Mb of *Pseudonocardia* sp. contigs, which accounts for an estimated 99.3% of its non-repetitive genomic content. This genome contains a limited array of carbohydrate catabolic pathways, but encodes for CO_2_ fixation via the Calvin cycle. The genome also encodes complete pathways for the catabolism of various trace gases (H_2_, CO and several organic C1 compounds) and the assimilation of ammonia and nitrate. We compared genomic content among related *Pseudonocardia* spp. and estimated rates of non-synonymous and synonymous nucleic acid substitutions between protein coding homologs. Collectively, these comparative analyses suggest that the community structure and various functional genes have undergone strong selection in the nutrient poor desert mineral soils and high-elevation atmospheric conditions.

## Introduction

The Atacama Desert is the driest and perhaps oldest desert on Earth, where an estimated 150 My of sustained aridity and 3–4 My of hyperaridity across the central plateau have shaped the landscape (Hartley et al., 2005). The Atacama region is bounded by the Andes to the east and by the coastal mountain range and the cold water Pacific Humboldt current to the west (Gómez-Silva et al., 2008). These barriers restrict the flow of atmospheric moisture, which in turn results in some of the most inhospitable proto-mineral soils on the planet that contain nearly undetectable organic carbon stocks and microbial biomass pools (Navarro-González et al., 2003). The eastern boundary of the region hosts large volcanoes that are situated in the leeward rain-shadow of the Andes. The upper plant-free reaches of these peaks are distinct from other more well studied Atacama geographic zones in that the higher elevation increases rates of precipitation, yet also increases rates of evaporation, sublimation, solar incidence and freeze-thaw cycling (Schmidt et al., 2009). Despite these additional stressors, the barren high volcanic deposits are a habitat still principally limited by water availability (Costello et al., 2009). Photo-atmospheric processes (e.g., lightning derived nitrate deposition, Michalski et al., 2004), likely play defining roles in these gravel-like mineral soils where biotic geochemical cycling is constrained to nearly undetectable levels.

Although meteorological data from the high-elevation reaches of the Atacama volcanoes are sparse (Richter and Schmidt, 2002), the restrictiveness of the conditions to biological activity is manifest in the biomass levels of the mineral soils, which are barely above detection limits, as well as microbial diversity estimates that rival the lowest ever sampled for exposed terrestrial systems (Costello et al., 2009; Lynch et al., 2012). The physical conditions that exclude nearly all microbial life seem to have been overcome by a limited spectrum of bacterial and fungal lineages that may have evolved the capacity for *in situ* activity. The most abundant of these organisms are Chloroflexi and certain Actinobacteria, mainly of the Actinomycetales, Acidimicrobiales and Rubrobacterales orders (Costello et al., 2009; Lynch et al., 2012).

Based on our initial molecular survey of these volcanic samples (Costello et al., 2009; Lynch et al., 2012), and work carried out in other areas of the Atacama where plant and microbial phototrophs are absent (Neilson et al., 2012), we hypothesized that chemoautotrophic microbes may be supplying organic carbon to simple and low-energy flux communities. Previous studies elsewhere have demonstrated the biological uptake of trace gases (CO and H_2_, but not CH_4_) in 26 year old plant-free and carbon limited Hawaiian volcanic deposits (King, 2003a), implying trace gases may be important energy sources where organic carbon accumulations are limited. The present metagenomic study was undertaken to develop a more comprehensive understanding of the potential metabolic traits, particularly focused on energy and nutrient acquisition, which the few community members found at the Llullaillaco Volcano study sites possess. The functional hypotheses developed through this study will be considered in light of the known environmental conditions present at these sites, and support the ongoing development of realistic growth conditions for culture based experiments.

Here we present a shotgun metagenomic study of a low-diversity and phylogenetically under-dispersed community, composed almost exclusively of Actinobacteria (>98% of all bacteria) found in the high-elevation (>6000 m elevation) Atacama Desert volcanic deposits. By leveraging the natural low diversity of these samples with deep coverage from long-read whole metagenome shotgun sequencing, we were able to characterize the genomic makeup of the community members at a high level of detail through reference database classification of raw sequence reads. Our high sequencing depth and coverage also enabled *de novo* assembly based analyses of selection through estimation of non-synonymous and synonymous mutation rates for protein coding genes of the most abundant community member's genome.

## Materials and methods

### Sample collection and preservation

Two snow free mineral soil samples located approximately 5 m apart were collected from the Llullaillaco Volcano (−24.718, −68.529) at an elevation of 6034 m above sea level (m.a.s.l.) during the austral summer in mid-February 2009. The top 4 cm of surface material, excluding rocks larger than 2 cm in diameter, were aseptically collected and frozen the same day in the field using blue ice packs. By the evening of the day the samples were collected, they were transferred to a −20°C freezer at the army barracks (on the Chile-Argentina border) near the field site. The next day they were driven (on ice in a cooler) to Salta, Argentina where they were again placed in a −20°C freezer until they were hand carried to Colorado in a thick-walled cooler on blue ice packs. They arrived in Boulder, Colorado within 24 h of being taken out of the freezer in Salta and were still frozen upon arrival (i.e., the ice packs hadn't melted). The samples have since been continuously stored at −20°C. Further details regarding these and other samples collected from the Llullaillaco Volcano can be found in Lynch et al. (2012).

### DNA extraction and sequencing and quality control

We utilized a modified serial silica filter binding protocol (Fierer et al., 2012) to overcome the low DNA yields of these low biomass samples and to avoid the potential biases introduced from random genomic amplification techniques. DNA extractions were quantified using PicoGreen dsDNA fluorometry (Thermo Fisher Scientific Inc.). We recovered 1 μg of gDNA from each of the samples, which required 10.4 g of volcanic debris from sample 1 and 4.8 g from sample 3 (Table 1). Negative extraction controls were run with the same batch of extraction reagents, but no soils were added. These negative control extractions were excluded from the sequencing libraries due to insufficient quantities of dsDNA. Samples were shipped to the Duke University Genome Sequencing and Analysis Core Resource where the long-read 454 GS FLX+ platform was used to sequence randomly fragmented bulk nucleic acid extractions.

**Table 1:** **Summary of sample characteristics for volcano metagenomes**.

**Sample**	**Mass for DNA extraction**	**16S RNAs**	**Observed OTUs**	**Shannon**	**Simpson**	**NRI (*p*-value)**	**pH**	**TOC**
1	10.4 g	363	78	5.146	0.954	4.99 (<0.001)	4.13	0.027
3	4.8 g	334	75	4.862	0.938	2.37 (0.01)	4.23	0.016

*The significant phylogenetic clustering of the microbial community is summarized by the positive Net Relatedness Index (NRI) of these low diversity low biomass communities. TOC, percent total organic carbon. pH and TOC values are from data and methods reported in Lynch et al. (2012)*.

Library parsing and removal of the 454 MIDs was achieved with the sfffiles package (454 Life Sciences) and manually confirmed using the Geneious (6.1.3) viewer. Reads were trimmed so they contained no more than five bases with quality scores of 15 or lower (Cox et al., 2010). Sequence length was required to be within two standard deviations of the mean length, and no more than five ambiguous bases per read were permitted. We found very low rates of artificial read duplication (Gomez-Alvarez et al., 2009, 0.31 and 0.13% for the sites 1 and 3 libraries respectively), which was tested using CD HIT (Fu et al., 2012), with settings 1 1 3 that require 100% sequence identity and length.

We used a 15-mer spectrum analysis (Supplementary Figure 2, Marçais and Kingsford, 2011) to visualize how sequencing depth relates to the total metagenomic complexity of the samples. Additional desert and non-desert metagenomes were downloaded from the MG RAST server (Meyer et al., 2008), ID 4446153.3 and all datasets from Fierer et al. (2012).

### rDNAs

A closed reference operational taxonomic unit (OTU) picking method (pick_closed_reference_otus.py, Caporaso et al., 2010) was applied to a UCLUST (Edgar, 2010) identified set of candidate 16S RNAs genes. This method overcomes the issue of sequencing different regions for the 16S rRNA gene with the shotgun technique. A 97% similarity was required for each candidate sequence alignment to the most current Green Genes reference dataset available (Release 13_5, McDonald et al., 2012). For the analysis of phylogenetic dispersion, near full length 16S rRNA gene sequences that have been previously published (JX098304—JX098810) were used to construct a maximum likelihood tree (Price et al., 2009) with the Green Genes reference dataset (13_5) clustered into 5088 OTUs_85%_. Phylocom 4.2 (Webb et al., 2008) was used to calculate a net relatedness index (NRI) value and associated one-tail *P*-values with 999 randomization iterations and the null hypothesis setting 2 (sample OTUs are drawn at random from the total species pool without replacement). This null hypothesis is intended to model the homogenizing effects of long distance atmospheric transport and deposition of bacterial cells from diverse sources, with a total absence of selection.

Fine scale phylogenetic trees were constructed with OTUs_1%_ of the full length 16S sequences determined by the QIIME pick_de_novo_otus.py workflow. SINA alignments (Pruesse et al., 2012) were built with Silva (115) reference database representatives (Quast et al., 2013) and maximum likelihood phylogenies were inferred with PhyML 3.0 (Guindon et al., 2010) using a GTR model of nucleic acid evolution.

### Genetic inventory

The SEED database (Overbeek et al., 2005) uses a hierarchical classification system where the broadest level (level 1) includes many anabolic and catabolic pathways and their associated single enzyme catalyzed intermediaries. Pairwise *t*-tests were used to calculate significance of gene category count differences (level 1) between the Llullaillaco Volcano libraries and a collection of desert and non-desert metagenomes, using the pooled SD option and a Bonferroni correction for multiple comparisons (α = 0.05/ (28 × 2) = 0.0009) in R (http://www.r-project.org/). Gene calls were made based on minimum ID of 60% and a maximum *e*-value of 1 e^−5^ for all BLAT alignments that were generated from MG RAST, and the SEED database.

### Assembly

*De novo* assembly was attempted on each of the two separate Llullaillaco site metagenomes with the MIRA V3.4.0 (Chevreux et al., 1999) signal trace assembly platform using the following settings: --job=denovo,genome,accurate,454 --highlyrepetitive --noclipping --notraceinfo --fasta -project=RL1All -SK:not=46 -AS:sep=yes 454_SETTINGS -ED:ace=yes -AL:mo=40:ms=30 -CL:bsqc=yes -LR:lsd=yes:ft=fastq. These settings require that each fragment addition to a contig have at least 40 high quality scoring bases of overlap and minimum quality scores of 30. They also restrict the variance of coverage levels across each contig to reflect the expectation that random shotgun sampling of each community member's genome should result in a unique coverage level that reflects its natural relative abundance in the community of genomes. This assembly approach assumes a theoretical copy number of one per unique genomic element leading to exclusion of repetitive elements, and also assumes that the main community members have significantly different relative abundances.

### Assembly evaluation and annotation

Tetramer based emergent self-organizing maps (ESOMs) http://databionic-esom.sourceforge.net/ were used to help evaluate contig binning (Dick et al., 2009) in conjunction with analysis coverage levels. Descriptions of the databionic ESOM settings and the Perl scripts used to calculate tetramer frequencies can be found at https://github.com/tetramerfreqs/binning. Consensus sequences from contigs were called with a majority rule to filter out all but the most abundant strains and low coverage ends were trimmed.

Bins of contigs that represent draft genomes and associated metadata were uploaded to the JGI IMG/ER database (Markowitz et al., 2012) for initial annotation. The phylogenetic origins of the JGI protein annotations were inspected and annotations for select coding DNA sequences (CDS) were checked manually. Completeness of the metagenome assembles was assessed by comparing protein family database (Punta et al., 2012) annotations to the list of conserved single copy genes (CSCGs, Rinke et al., 2013). Putative genes involved in major metabolic pathways were manual curated by evaluating blastx alignments and through literature-based refinement of functional annotations.

### Comparative genomics and analysis of selection

Clusters of orthologs genes (COGs, Tatusov, 1997) for the three publically available *Pseudonocardia* sp. genomes were downloaded from the IMG/ER database. COG count data were subjected to hierarchal centroid clustering with Cluster 3.0 http://bonsai.hgc.jp/mdehoon/software/cluster/software.htm. and visualized with heatmaps drawn in TreeView (Saldanha, 2004).

Even when genes share clear homologous relationships they may perform divergent functions. One way to detect the signature of divergent selection between orthologous genes is through the comparison of rates of non-synonymous (K_a_) to synonymous (K_s_) mutations. When selection is weak or absent K_a_:K_s_ratios should be close to one since genetic drift should have an equal chance of causing either non-synonymous or synonymous mutations. However, when divergent selection drives altered amino acid coding potential, rates of non-synonymous mutations should be elevated relative to synonymous mutations (Yang, 1998). A Perl pipeline was used to link the following steps together for an iterative K_a_:K_s_ analysis. Pairs of candidate CDS orthologs between our best volcano *Pseudonocardia* sp. draft genome and the *Pseudonocardia asaccharolytica* (IMG ID 13496) draft genome were identified as reciprocal blastn hits (with ≥70% identity for 100 bp). Protein guided DNA alignments were generated for each CDS pair through the TranslatorX approach (Abascal et al., 2010), which relies on Muscle (Edgar, 2004) to align predicted amino acid sequences. Codeml (PAML 4.7, Yang, 2007) was then used to estimate rates of non-synonymous (K_a_) and synonymous (K_s_) nucleic acid substitutions for each ortholog pair alignment, using the WAG model of amino acid evolution. Ortholog pairs found with signatures of positive selection for amino acids substitutions (K_a_:K_s_ ratios of ≥ 1) were checked manually and annotated with a database of genes from the *P. asaccharolytica* draft genome using blastx.

### Hydrogenase phylogenetics

To place the [NiFe]-hydrogenase genes from our volcano *Pseudonocardia* sp. assembly into a broader phylogenetic context, we constructed a phylogeny using available sequence data from other studies. A broad sampling of [NiFe]-hydrogenase large subunit amino acid sequences was obtained from the list of sequences provided by Vignais and Billoud (2007), along with their subgroup annotations. Sequences for a fifth subgroup were obtained through blastn searches using our assembled sequence, as well as from Constant et al. (2010). Incomplete sequences were not included in our analysis. All amino acid sequences were aligned using ClustalW2 (Larkin et al., 2007) using default parameters, and a phylogeny was made using the neighbor-joining algorithm implemented in MEGA 6 (Tamura et al., 2013) using the Poisson model with 1000 bootstrap replications.

## Results

### Sequencing and rDNA diversity

**Figure 1:**
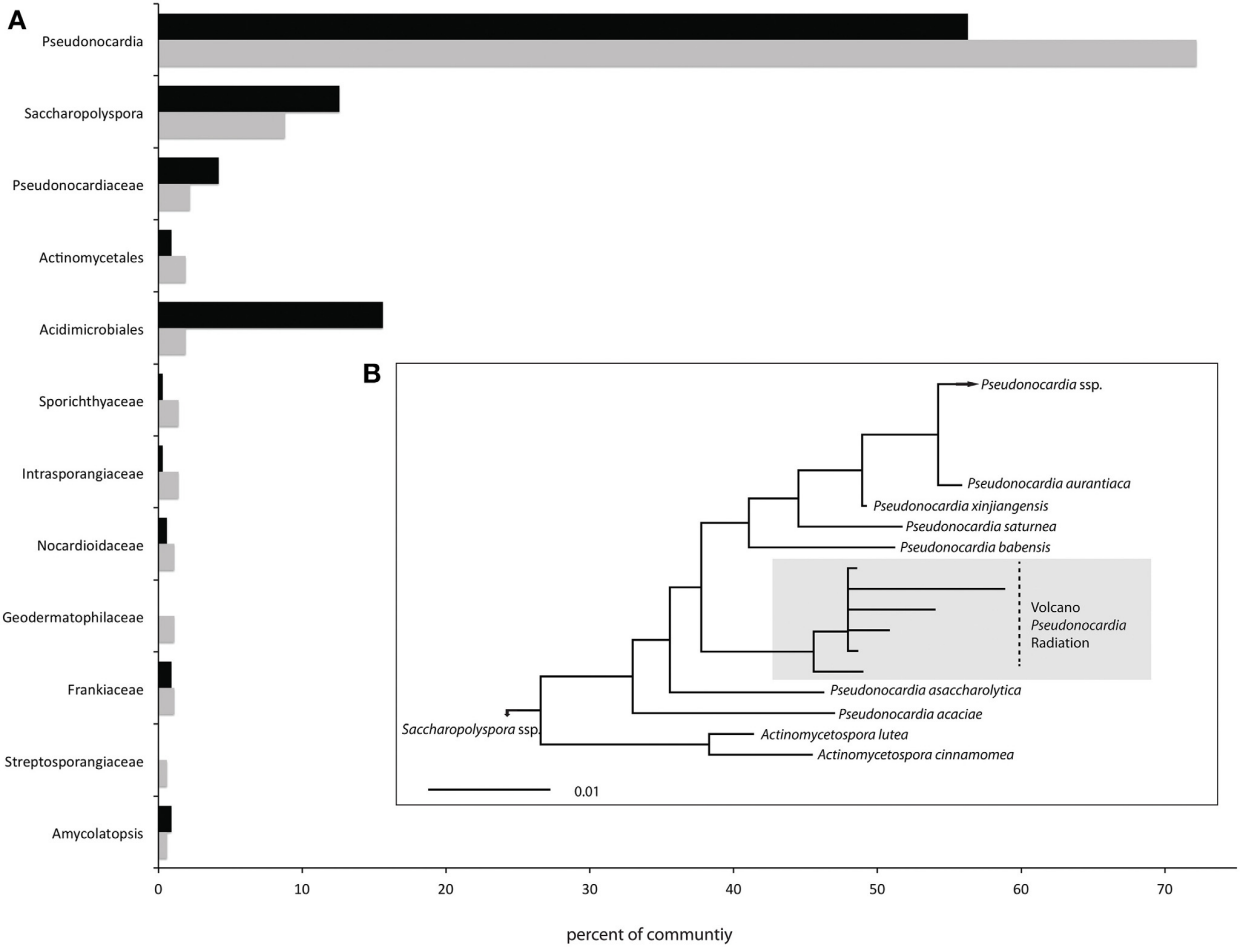
**Community profile (A) All OTU_3%_ taxonomic assignments from each site that represent at least 1% of the total metagenome 16S gene reads**. These 12 OTUs constitute 94.3 and 92.6% of the total 16S gene reads from sites 1 (gray bars) and 3 (black bars) respectively and are all members of Actinomycetales other than the single Acidimicrobiales OTU_3%_. **(B)** Maximum likelihood phylogeny of the most abundant Pseudonocardia OTU_3%_, split into sub OTUs_1%_. The scale bar represents 1% divergence between nucleic acid sequences.

After trimming we were left with 3.85 million reads that total 1.3 Gb of DNA sequence data for downstream analysis. Each of the two site libraries contained nearly identical distributions of bacterial (99.2%), eukaryotic (0.5%) and archaeal (0.3%) reads, based on all MG RAST annotation databases. We found a low diversity community populated mostly by Actinobacteria (Table 1), which make up 98.6 and 97.9% of the 16S rRNA genes from the site 1 and 3 libraries, respectively. This highly uneven community structure is significantly under dispersed (*P* < 0.001 and 0.01 for the phylogenetic randomization tests on the two samples), indicating a likely non-random assemblage of bacterial lineages. All lineages shown in Figure 1 belong within the Actinomycetales, other than an OTU_3%_ belonging to the Acidimicrobiales order (Supplementary Figure 1) that makes up 15.6% of the site 3 library, but only 1.9% of the site 1 library. The *Pseudonocardia* are by far the most abundant lineages (72.2% of site 1 and 56.3% of site 3 total 16S reads) and the *Saccharopolyspora* (Pseudonocardiaceae) also make up 8.8% and 12.6% of total 16S rRNA gene reads from sites 1 and 3, respectively.

### Genetic inventory

**Figure 2:**
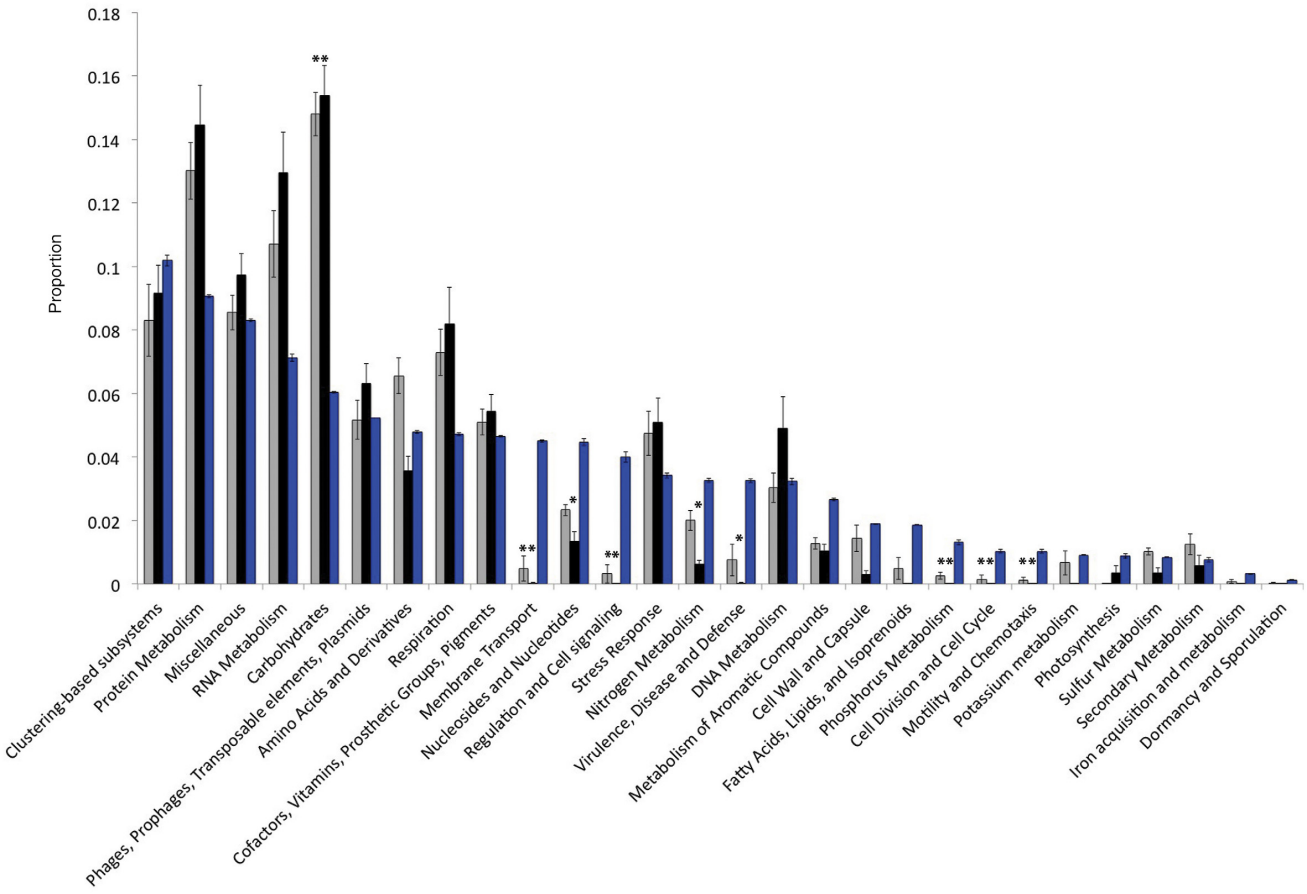
**Inventories of gene functional categories, comparing non-desert (gray), desert (black) biomes to the high-elevation volcano metagenomes (blue)**. Asterisks indicate Bonferroni corrected significant differences (*P* < 0.0009) between the volcano data and desert or non-desert data (desert to non-desert comparisons not shown) for all pairwise *T*-tests.

The Llullaillaco metagenomes show a pronounced reduction in genes associated with carbohydrate metabolism compared with other desert and non-desert metagenomes (Figure 2). By contrast we found significant enrichment of pathways categorized as membrane transport, nucleotide metabolism, regulation and cell signaling, nitrogen metabolism and virulence and defense. Examining the presence and absence of metabolic pathways within the total metagenome, we found no evidence for complete photosynthetic pathways, yet found complete gene sets for the oxidation of CO and H_2_, and for CO_2_ fixation with the Calvin cycle. Methylotrophic pathways also suggest a role for other C1 compound oxidation and assimilation including: methanol, formaldehyde, formate and perhaps methane. No nitrogen (N_2_) fixation or ammonia monooxygenase genes were identified, but genes for nitrate (NO^−^_3_) reduction (nitrate reductase) and ammonia (NH_3_) assimilation (glutamine synthetase) were found in high abundance.

### Genome assembly results

We were able to assemble and bin contigs (Supplementary Figures 3, 4) that represent composite genomes of the most abundant *Pseudonocardia* sp. (Table 2), as well as the other lower abundance community members, such as a member of the Acidimicrobiales (Supplementary Figures 1, 3). The best *Pseudonocardia* assembly appears to represent a nearly complete set of non-repetitive genomic elements since it contains 138/139 CSCGs (missing a DNA uptake competence gene, PF03772). None of the CSCGs were present in more than one copy in the metagenome assemblies, suggesting we did not greatly over-assemble this genome. The CSCGs are 139 protein coding genes that were found to occur only once in at least 90% of the 1515 finished bacterial genomes available in the IMG/ER database (Rinke et al., 2013). Within each of the new *Pseudonocardia* sp. assemblies, 2–3 single nucleotide polymorphisms (SNPs) were present in many of the CDS regions, which are likely indicative of strain and population level variation.

**Table 2:** **Summary of metagenome *Pseudonocardia* sp. assemblies and nearest phylogenetic reference genome, *P. asaccharolytica* (JGI IMG id 13496)**.

**Genome**	**Ave. coverage**	**Size**	**Max. contig**	**Contigs**	**CDS**	**CSCGs**	**GC %**	**rRNA operons**
Site 1 volcano *Pseudonocardia* sp.	68–115	4.87 Mb	92,460 bp	336	5434	138/139	70.7	3
Site 3 volcano *Pseudonocardia* sp.	45–68	4.63 Mb	117,339 bp	332	5055	129/139	70.8	3
*Pseudonocardia asaccharolytica*	Na	5.05 Mb	441,096 bp	72	5024	Na	71.7	3

*Conserved single copy genes (CSCGs) ratio estimates the completeness of the non-repetitive components of the metagenome assembles*.

### COG comparisons

**Figure 3:**
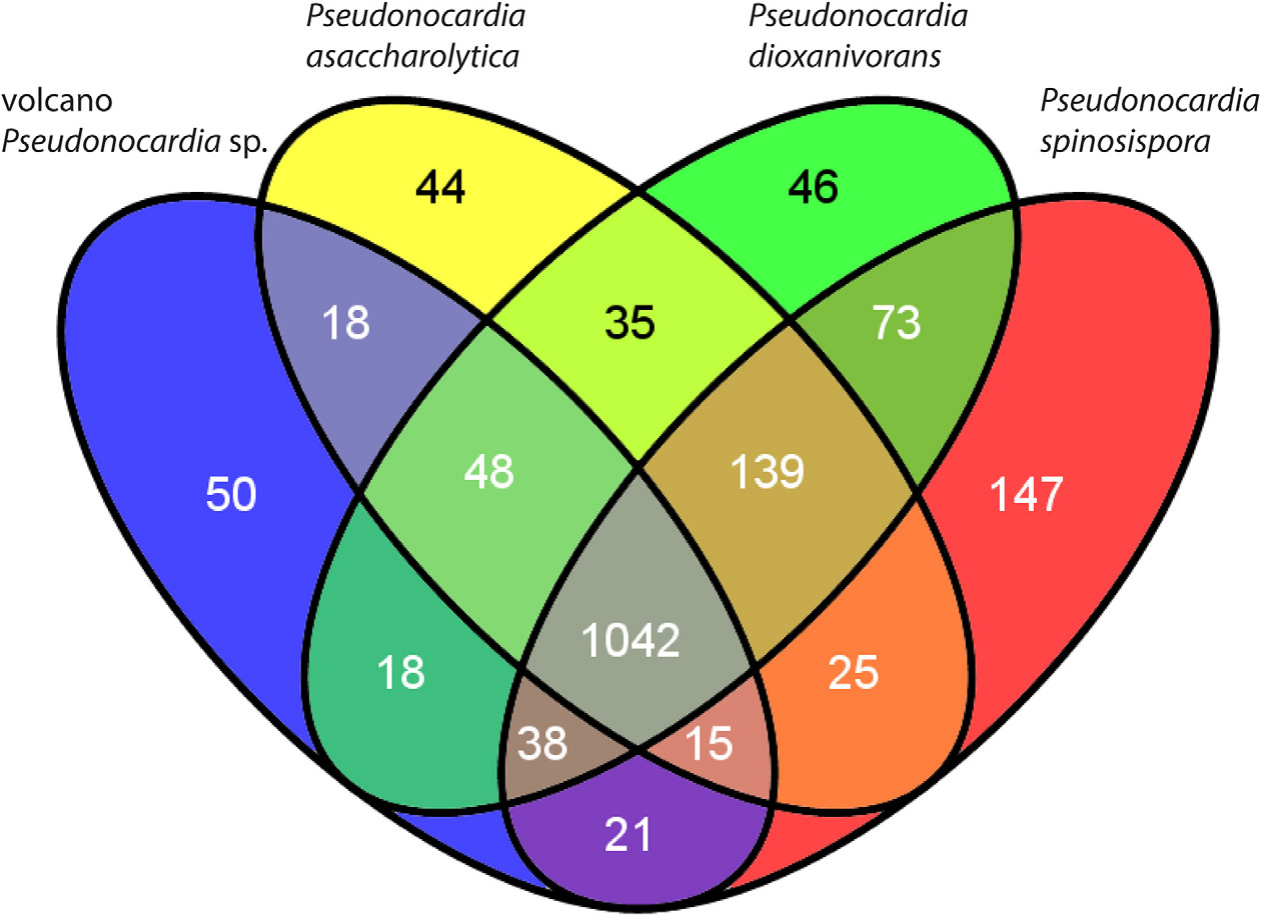
**Venn diagram of the shared and unique genes (COGs) among named *Pseudonocardia* spp. with complete genomes and the volcano *Pseudonocardia* sp. genome assembly**. Although most of the 50 COGs unique to the volcano *Pseudonocardia* sp. (Supplementary Table 1) are classified as “function unknown” or “general function prediction only,” the six additional defense mechanism related COGs and the nine fewer carbohydrate transport and metabolism COGs in the volcano *Pseudonocardia* sp. stand out as potentially relevant functional differences with other *Pseudonocardia* spp.

COG counts from our highest quality *Pseudonocardia* sp. assembly (68–115 × coverage bin from site 1) and the three other publicly available genomes for named *Pseudonocardia* spp. (Supplementary Table 1, Figure 3) highlight some of the specific differences in genome content. We found certain COGs like those needed for CO oxidation are conserved at high copy numbers across all the *Pseudonocardia* spp., and that COGs such as those required for assimilatory nitrate reduction and carbon fixation (RuBisCO) show relatively higher counts in both our metagenome assembly and *P. asaccharolytica*. Other highly abundant gene clusters within our metagenome assembly bear resemblance to the more phylogenetically distant *Pseudonocardia* spp. These clusters include the antibiotic producing non-ribosomal peptide synthesis pathway (NRPS), various ABC peptide importers, cytochrome P450 monooxygenase, and several recombinases.

### Signatures of selection analysis

**Figure 4:**
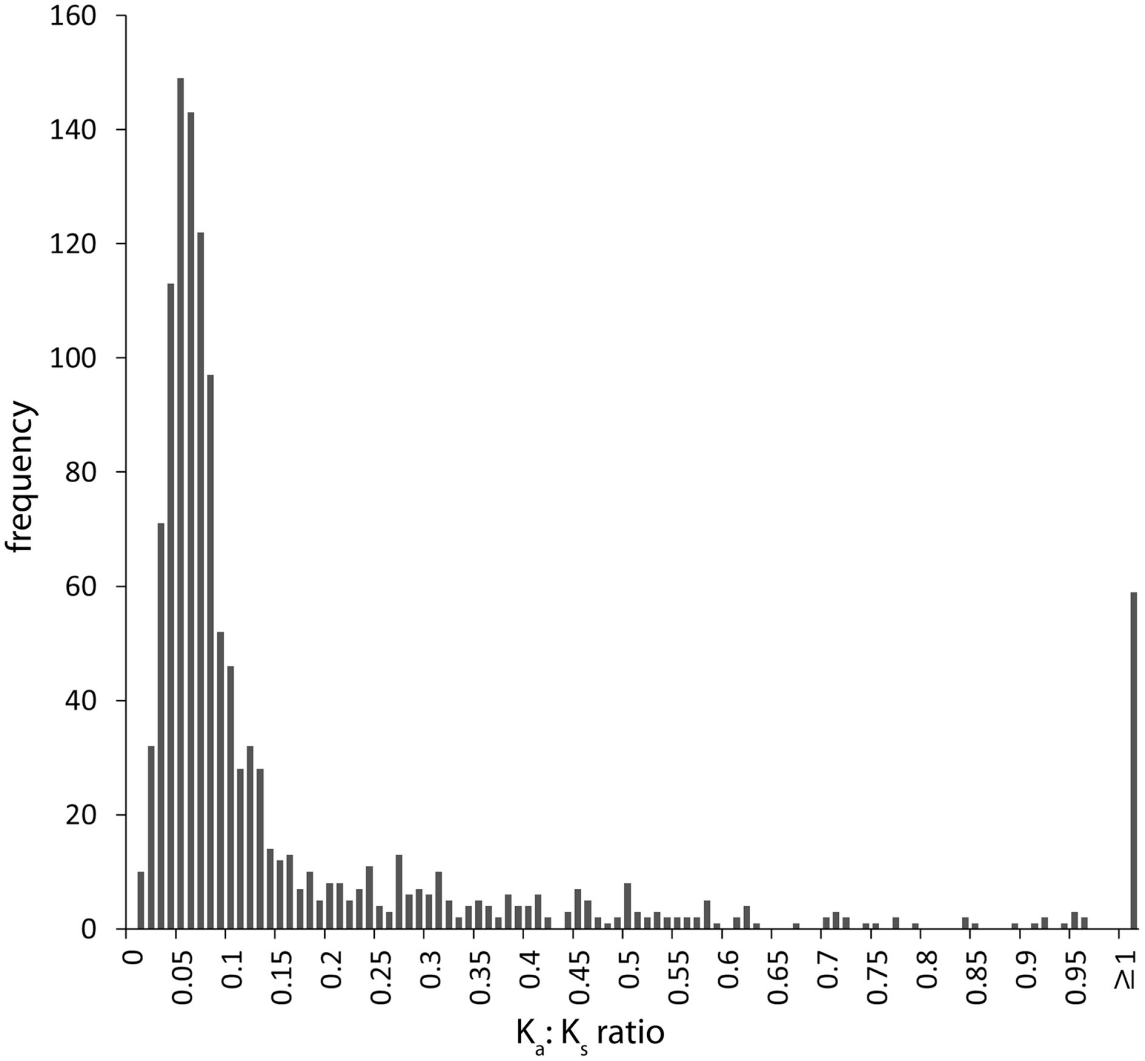
**Distribution of K_a_:K_s_ ratios for 1260 pairwise orthologous protein coding sequences between the best volcano *Pseudonocardia* sp. assembly and its closest fully-sequenced relative, *Pseudonocardia asaccharolytica*, showing the majority of genes (95.3%) to be under purifying or relaxed selection regimes, where synonymous substitutions that do not alter the amino acid coding potential dominate the gene**. However, some outliers (4.7%) display higher levels of non-synonymous mutations (≥1 K_a_:K_s_) likely driven by divergent selection from the harsh high-elevation desert conditions. This analysis was limited to 23% of total volcano *Pseudonocardia* sp. genes due to the high degree of overall genomic divergence between these two species.

Of the 5024 annotated CDS from the draft *P. asaccharolytica* genome we were able to initially align 1722 orthologous coding sequences from our best metagenome *Pseudonocardia* sp. assembly with at least 70% nucleotide identity. Of these, manual inspection filtered out 462 gene pairs that were poorly aligned or were not true homologs across the entire sequence. There were 59 remaining ortholog pairs (4.7%) with estimated K_a_:K_s_ ratios ≥ 1, which reflects elevated rates of non-synonymous mutations brought about through strong divergent selection acting upon the amino acid sequences (Figure 4, Supplementary Table 2).

### Characteristics of the volcano *Pseudonocardia* sp. genome

**Figure 5:**
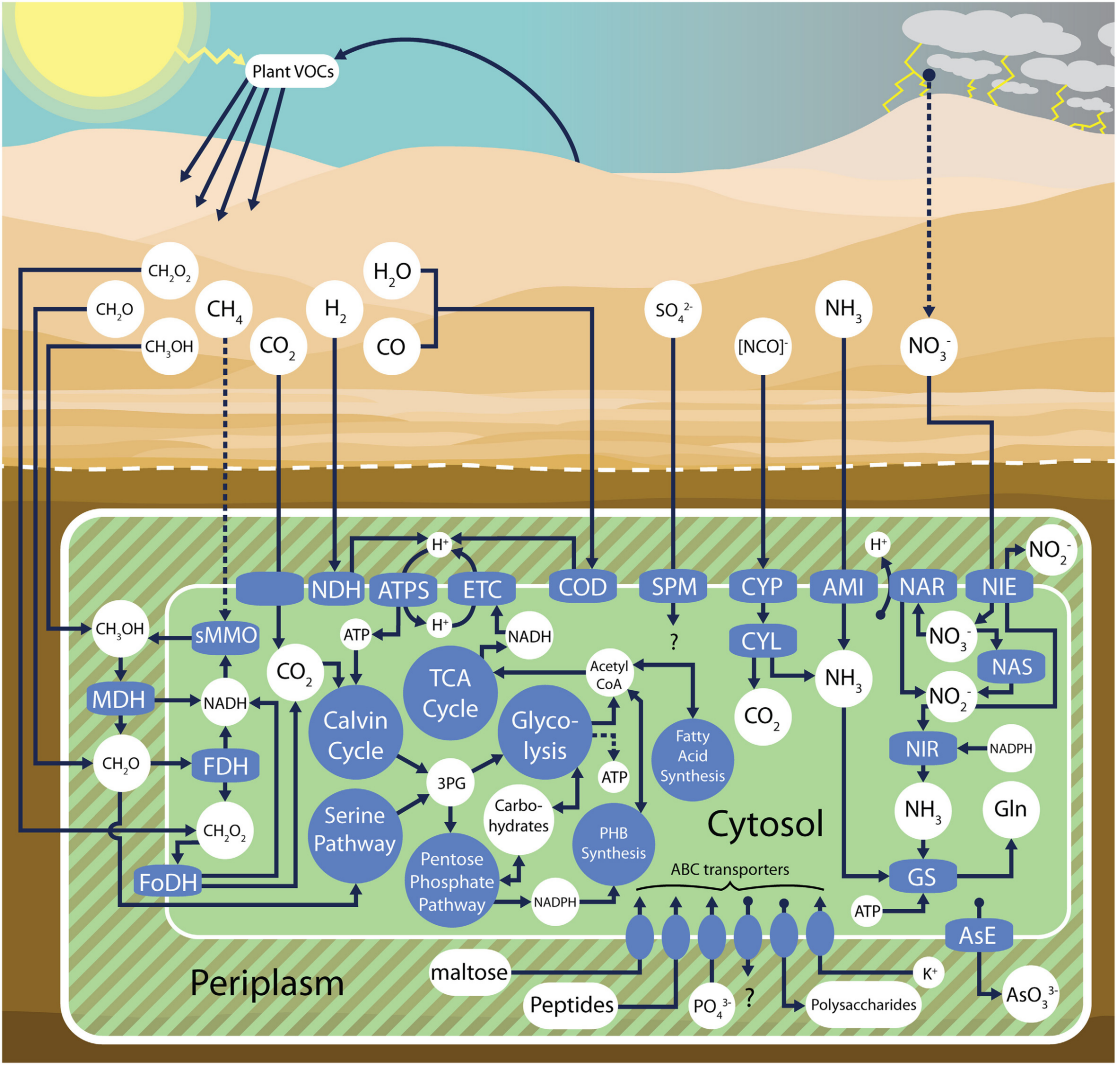
**Ecophysiological overview of the volcano *Pseudonocardia* sp. metabolic pathways as inferred from assembled metagenomic data**. sMMO, soluble methane monooxygenase; MDH, (PQQ)-dependent methanol dehydrogenase; FDH, formaldehyde dehydrogenase; FoDH formate dehydrogenase-O; NDH, group 5 high-affinity NiFe hydrogenase, ATPS, ATP synthase; ETC electron transport chain; COD, form I carbon monoxide dehydrogenase; AsE, arsenite efflux; CYP, cyanate permease; CYL cyanate lyase; AMI, ammonium importer; NAS, assimilatory nitrate reductase; NAR, respiratory nitrate reductase; NIE, nitrite extrusion protein; NIR, nitrite reductase; GS, glutamine synthetase; SPM, sulfate permease; 3PG 3-phosphoglyceric acid; PHB, polyhydroxybutyrate; Gln, glutamine.

The volcano *Pseudonocardia* sp. genome is at least 4.9 Mb (Table 2) and contains many of the pathways that define the total community metabolic potential (e.g., aerobic heterotrophic metabolism, NO^−^_3_ and NH_3_ utilization, H_2_ and CO oxidation, CO_2_ fixation and methylotrophic pathways, Figure 5). Many genes (33%) were found with multiple copies in the genome, suggesting a possible role for gene duplication events during the divergence of this genome. Potential carbohydrate oxidation pathways are quite limited, with genes present only for the utilization of glucose, mannose, ribose, gluconate, maltose, trehalose, lactose, and galactose that feed into the Embden-Meyerhof-Parnas pathway or the pentose phosphate pathway. Carbohydrate uptake potential is apparently even more restricted as only a single annotated maltose ABC importer was identified. A complete list of putative gene annotations can be found in the IMG/ER database (id 45716).

### Hydrogenase phylogeny results

**Figure 6:**
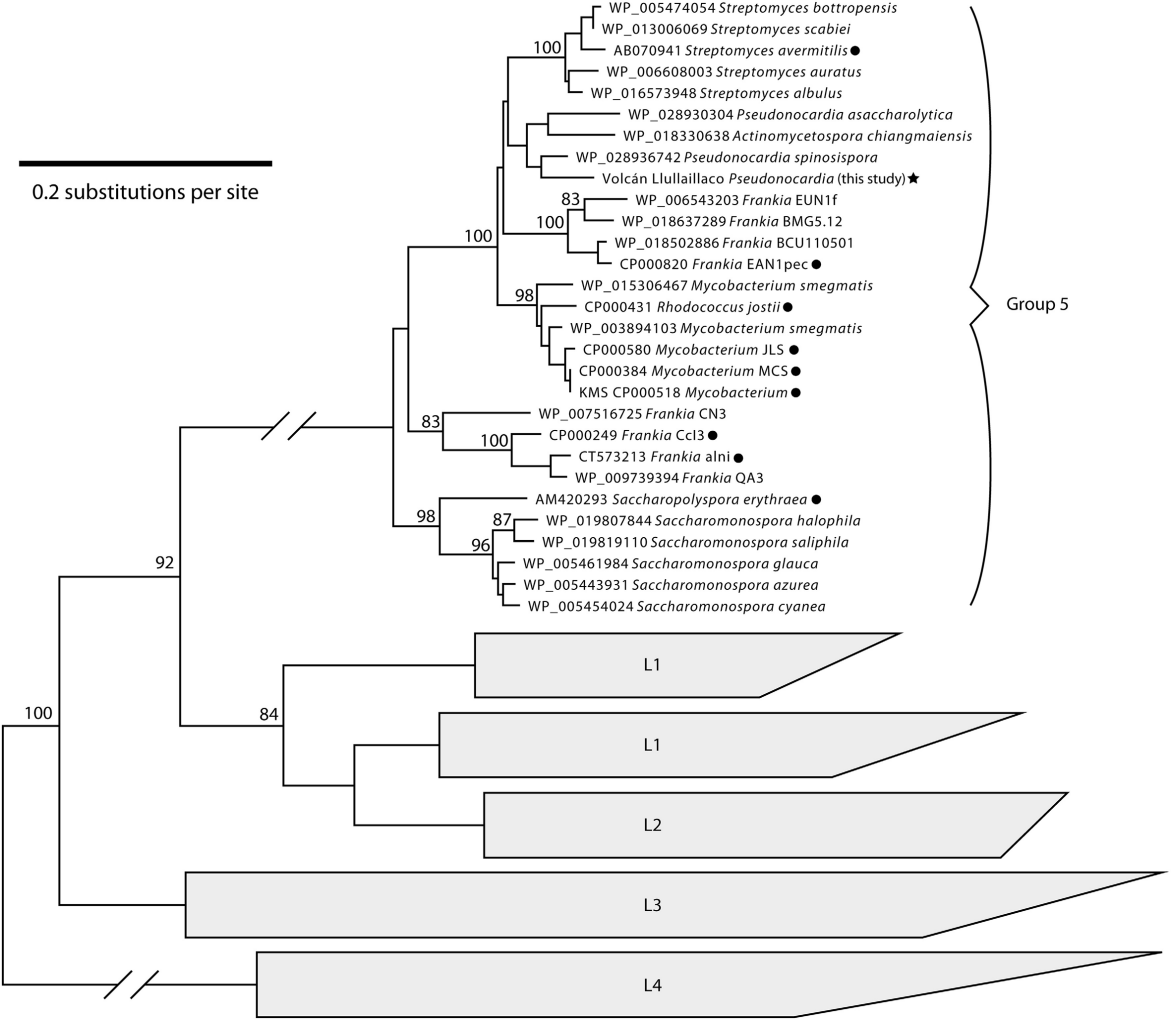
**Neighbor-joinging phylogenetic tree of [NiFe]-hydrogenase amino acid sequences**. The phylotype from our *Pseudonocardia* sp. assembly (star) falls into the same clade as sequences shown in Constant et al. (2010), which are marked with circles. Sequences from other [NiFe]-hydrogenase large subunit subclades (L1–L4, Vignais and Billoud, 2007) are shown as the outgroup. Bootstrap support values are shown for nodes present in over 80% of bootstrapped trees. The scale bar represents 20% divergence between amino acid sequences.

Our phylogenetic analysis of [NiFe]-hydrogenase sequences confirmed that the volcano *Pseudonocardia* sp. assembly includes a group 5 [NiFe]-hydrogenase gene (Figure 6). Our phylogeny resolved a monophyletic clade for hydrogenase group 5, which includes the group 5 hydrogenase sequences from Constant et al. (2010) as well as several other Actinobacterial phylogypes. [NiFe]-hydrogenase protein sequences that are most closely related to the volcano *Pseudonocardia* sp. came from *P. asaccharolytica*, *Pseudonocardia spinosispora*, and *Actinomycetospora chiangmaiensis*.

## Discussion

The conditions present in the most extreme Atacama Desert soils exclude most life and leaves open the questions of if and how microbes may survive there. Previous studies of Atacama Desert soil microbiota have used either 16S gene based culture-independent approaches (Navarro-González et al., 2003; Costello et al., 2009; Lynch et al., 2012; Neilson et al., 2012), or to a limited extent culture-dependent methods (Lester et al., 2007; Okoro et al., 2009). Taken together, the pioneering work done on Atacama soils indicates that low diversity microbial communities are present at many sites, though few details have emerged regarding the origins and functional nature of these microorganisms. In this study, we used a deep metagenomic sequencing strategy to examine the structure and functional potential of the Llullaillaco Volcano microbial community (Lynch et al., 2012). Difficulty with extracting DNA from very low biomass mineral soils required us to pool roughly the equivalent of 60 standard 0.25 g soil DNA extractions to achieve the quantity of genomic DNA necessary for shotgun metagenomic sequencing. As a result, this dataset is less spatially expansive than our previous amplicon based analysis (Lynch et al., 2012), yet still demonstrates the low-diversity community structure extends throughout a relatively large volume of soil. Despite the limitations of this study, the approach allowed for a more thorough description of the Llullaillaco Volcano microbial community structure, and provides an initial insight into the protein coding potential of the metagenome as well as the most abundant community member's genome.

Through this approach we found an extremely low-diversity community of organisms (Figure 1, Table 1) that host an unusual inventory of functional genes (Figure 2), including an absence of phototrophic pathways and limited capacity for heterotrophic carbohydrate metabolism. The low diversity community lacks many of the clades previously recovered from high-elevation air (Bowers et al., 2012) and dust (Stres et al., 2013) microbiome studies, suggesting a high degree of environmental selection that could occur during atmospheric transport to these Atacama sites, or during active or dormant residence in the mineral soils.

The most abundant 16S gene OTU (*Pseudonocardia* sp.) recovered from the two sites used in this study (and from the third “low site” from Lynch et al., 2012), shares a relationship with *Pseudonocardia* sp. detected in other high elevation samples from Himalayan and Antarctic mineral soils (Rhodes et al., 2013), as well as with isolates from Icelandic volcanic deposits (Cockell et al., 2013) leaving open the possibilities it may be native to these sites or that it could be present at the Llullaillaco Volcano sites as a consequence of atmospheric transport (Stres et al., 2013). It is noteworthy that the Acidimicrobiales OTUs_3%_ (Figure 1) found in this environment (15.6% of the site 3 library, and 1.9% of the site 1 library) is related to known inhabitants of fumaroles (Supplementary Figure 1, Benson et al., 2011; Itoh et al., 2011), so it is likely that at least some of the organisms present at our research sites are the result of regional wind transport from active fumaroles on nearby Socompa Volcano (Costello et al., 2009), or from as yet undiscovered fumarolic activity on Llullaillaco Volcano. Indeed, we found Acidimicrobiales 16S gene sequences identical to those from the Llullaillaco Volcano in warm fumaroles of Socompa Volcano (Costello et al., 2009). It is also possible that the presence of known fumarole inhabitants indicates that our research sites are located on soils that were originally fumarolic and that the organisms found there are relics that have survived as dormant spores. This would explain the presence of genes for the utilization of gases that are found in fumarolic emissions (e.g., CO and H_2_), rather than the idea that they serve to metabolize the exceedingly low concentrations of atmospheric gases found at elevations above 6000 m.a.s.l.

### Energetics

Detailed examination of the most abundant community member's genome assembly reveals unique genetic content (Figure 3), evidence for divergent natural selection acting on certain homologs (Figure 4, Supplementary Table 2) and complete metabolic pathways related to trace atmospheric substance metabolism (Figure 5). Unidentified soil oligotrophs have long been suspected of oxidizing ubiquitous trace gases like H_2_, CO, and CH_4_ based on evidence from bulk soil process studies (Conrad, 1996; Constant et al., 2011). Although unequivocal demonstrations of bacterial growth and cell division from trace gas metabolism have been elusive, several actinobacterial isolates have been shown to oxidize ambient H_2_ and CO at atmospheric concentrations (Constant et al., 2008; King, 2003b). In certain actinobacteria, ambient H_2_ oxidation has now been conclusively tied to the activity of high-affinity group 5 [NiFe] hydrogenases (Greening et al., 2014).

[NiFe] hydrogenases are membrane-bound enzymes that catalyze the splitting of periplasmic H_2_, facilitating the production of a proton gradient for ATP synthesis (Figure 5, “NDH”). A novel group 5 [NiFe] hydrogenase gene set is present in our genome assembly of the most abundant volcano *Pseudonocardia* sp. (Figure 6), indicating that the dominant organism at this site likely has the ability to utilize atmospheric concentrations of H_2_ (0.53 ppmv, at sea level, but about 0.24 ppmv at 6000 m.a.s.l.) for energy production. Greening et al. (2014) also found that *Mycobacterium smegmatis* group 5 [NiFe] hydrogenase expression levels increased under carbon starvation conditions, implicating the oxidation of H_2_ as a source of electrons during low metabolic states. Given the low levels of organic carbon measured at the volcano sites (Table 1), and the phylogenetic affiliation between the group 5 volcano *Pseudonocardia* sp. [NiFe] hydrogenase and the *M. smegmatis* group 5 [NiFe] hydrogenase (sharing 80% amino acid identity) studied by Greening et al. (2014), oxidation of trace H_2_ seems to be a plausible energy source for the new *Pseuodnocardia* sp. However, [NiFe] hydrogenase genes are not the only genes we observed that could be used to metabolize atmospheric substrates.

Previous studies have correlated a widespread occurrence of carbon monoxide dehydrogenase genes with soil CO uptake (King, 2003a; Weber and King, 2010; Quiza et al., 2014), and various soil bacterial isolates have been confirmed to oxidize CO at atmospheric concentrations (<400 ppbv at sea level, Hardy and King, 2001; King, 2003b). Carbon monoxide dehydrogenase functions similarly to [NiFe] hydrogenase, in that it is a membrane-bound enzyme that facilitates the generation of a proton gradient. In this case, the enzyme oxidizes CO and reduces H_2_O, forming CO_2_ and two periplasmic protons (Figure 5, “COD”). *M. smegmatis* has been shown to be capable of trace CO uptake, and hosts canonical type I carbon monoxide dehydrogenase genes (Quiza et al., 2014), similar to the CO dehydrogenase genes present in the volcano *Pseudonocardia* sp. assembly. However, it is not yet clear how this activity affects cellular physiology. It is likely that tropospheric CO oxidation is often a supplemental energy source, contributing to a mixotrophic metabolism (King and Weber, 2007). Thus, physiological work focused on high-affinity CO oxidizing bacteria must carefully consider the possible requirements and roles of organic carbon sources, in addition to tracking low-concentration CO uptake (King and King, 2014).

The volcano *Pseudonocardia* sp. genome encodes complete pathways for the oxidation and assimilation of methanol, formaldehyde, and formate (Figure 5). The atmosphere contains very low concentrations of these gases mainly due to plant volatile emission and photochemical reactions (Hu et al., 2011; Stavrakou et al., 2011; Luecken et al., 2012). The study of bacterial metabolism of atmospheric concentrations of these C1 compounds is limited, although efforts are underway to develop an understanding of the distributions of methylotrophs and how they influence the global methanol cycle (Kolb and Stacheter, 2013). Furthermore, some evidence suggests that various Actinobacteria (e.g., *Streptococcus* and *Rhodococcus* spp., Yoshida et al., 2007) are capable of “CO_2_ dependent oligotrophic growth” under laboratory carbon starvation conditions by oxidizing ambient methanol and formaldehyde (Yoshida et al., 2011), suggesting these C1 gases can be atmospheric sources of energy and carbon for some bacteria.

Methane is the most abundant of the trace gases at 1.79 ppmv (or 0.80 ppmv at 6000 m.a.s.l.), so would seem to be a likely target for trace gas oxidizers. However, the Llullaillaco Volcano metagenome lacks any identifiable particulate methane monooxygenase (pMMO) genes, which have been previously identified as likely coding for the high-affinity methane oxidation enzymes in various soils (Bull et al., 2000; Kolb, 2009). Likewise the study of early-successional Kilauea Volcano soils by King (2003a) detected CO and H_2_ uptake, but not CH_4_. Yet the volcano *Pseudonocardia* sp. does encode all genes required for a putative iron-dependent soluble methane monooxygenase (sMMO) enzyme that could function to oxidize methane to methanol, which would then be fed into the abovementioned methylotrophic pathways. sMMOs are notoriously non-specific enzymes (Green and Dalton, 1989), and atmospheric concentrations of methane have not yet been reported to support bacterial growth (Theisen and Murrell, 2005; Conrad, 2009). Nevertheless, the evidence for widespread ambient methane oxidation (McDonald et al., 2008) and experimental confirmation of methane oxidation by members of the phylum Verrucomicrobia (Dunfield et al., 2007) illustrates the continued need to explore the phylogenetic and geographic distributions of methane oxidizers.

Given the presence of these various gas utilization pathways in the volcano *Pseudonocardia* sp. genome (Figure 5), and the constant availability of these substrates at low concentrations in the atmosphere, the high-elevation volcanic deposit community may rely on a mixture of diffuse atmospheric substrates in the absence of direct photosynthetic inputs to at least maintain redox balance, or perhaps even to drive carbon fixation. However, it is important to note the volcano *Pseudonocardia* sp. shares nearly all of these aforementioned trace gas oxidation pathways (Figures 5, 6) with *P. asaccharolytica*, its nearest phylogenetic relative (Figure 1). *P. asaccharolytica* does lack a (PQQ)-dependent methanol dehydrogenase gene, but these were present in other *Pseudonocardia* spp. (Figure 3). While no studies to date have tested *P. asaccharolytica* for trace gas metabolism either *in situ* or in culture (Reichert et al., 1998), the trace gas metabolism related genes common to the *P. asaccharolytica* and the volcano *Pseudonocardia* sp. genomes have been shown to confer trace gas metabolism capacity in other bacteria (Figure 6), making it a plausible trait shared by various members of this genus. Consequently, the relevance of trace gas utilization as a potential metabolic strategy in the harsh Atacama Desert mineral soils of this study is difficult to interpret, since trace gas metabolism genes are not exclusive to *Pseudonocardia* sp. recovered from desert environments.

Atmospheric gas metabolism is not mutually exclusive with other trophic strategies. The volcano *Pseudonocardia* sp. hosts fully encoded aerobic heterotrophic and autotrophic carbon acquisition pathways, and several energy storage pathways (Figure 5). The large and small RuBisCO subunit genes of the volcano *Pseudonocardia* sp. both cluster within the form IC clade, which contains other known bacterial facultative autotrophs (Yuan et al., 2012) including various Actinobacteria such as *P. asaccharolytica*, further suggesting a flexibility in carbon and energy acquisition physiology. It is certainly possible this organism is opportunistic, capable of survival at low metabolic rates through the utilization of a variety of low-concentration and constantly replenished atmospheric gases, but perhaps is also capable of capitalizing on pulses of other multi-carbon nutrients and water when they become available, such as after a snow melt event. Further understanding of the environmental conditions and how they vary through annual cycles at these difficult to access field sites combined with direct experimental growth assays will be required to test if and how this bacterium, or other members of the community, may grow under and respond to, variable and stressful conditions.

### Stress tolerance and other traits

Metabolism of various trace atmospheric substrates may be important adaptations to survival in the harsh and nutrient limited desert volcano environment, but the reduced and under-dispersed phylogenetic diversity of the microbial community (Figure 1, Table 1) suggests that other traits must be important for fitness, given that H_2_ and CO oxidizing genes are present in many species of several bacterial phyla. Actinobacteria have a seemingly ubiquitous distribution across varied terrestrial and aquatic environments (Dinsdale et al., 2008), but are relatively most abundant in cold-desert soil environments (Fierer et al., 2012). Some obvious traits of the actinobacteria are likely linked to desert fitness, such as gram positive cell wall architecture, which is perhaps an original adaptation to ancient terrestrial colonization (Battistuzzi and Hedges, 2009; Rinke et al., 2013), and the ability of many lineages to sporulate. However, given the metabolic diversity and rapid genomic evolution found within this phylum (Zaneveld et al., 2010), the full scope of desert actinobacteria traits remains largely uncharacterized.

The volcano *Pseudonocardia* sp. assembly contains COGs with relatively high copy number compared to other species of the genus that could possibly underlie stress tolerance adaptations including: DNA replication and repair machinery, transcriptional regulators, response regulators, cytochrome P450, arabinose efflux permeases, ABC-type multidrug transport systems and non-ribosomal peptide synthesis pathways (NRPS, Supplementary Table 1). It is not possible to determine the exact functional roles these genes play without experimental confirmation, but it is conceivable they could be linked to adaptations to the stresses of wet-dry or freeze-thaw cycling or UV exposure. The multiple copies (≥18) of the NRPS genes are notable because they share sequence homology most similar to the antibiotic gramicidin D gene set (Kessler et al., 2004). Considering the known importance of extrapolymeric substance production as a xerotolerace trait for many microorganisms (Lennon et al., 2012), and the presence of arabinose and polysaccharide export genes in the volcano *Pseudonocardia* sp. genome, it is not surprising that investment in antibiotic defense mechanisms that may ward off scavengers of these vulnerable carbon sources (e.g., fungi, Schmidt et al., 2012) may also be necessary.

We compared all well aligned homologs between the volcano *Pseudonocardia* sp. to *P. asaccharolytica* in order to identity how selection may have affected the amino acid sequences (and functions) of certain genes. *P. asaccharolytica* was isolated from a dimethyl sulfide and tree-bark biofilter enrichment experiment (Reichert et al., 1998), but little else is known about its ecology or physiology other than the lack of ability to oxidize any of the single carbohydrates tested in the original report, and that it can be grown at moderate rates on TSA media at mesophillic temperatures. Our analysis identified 59 volcano *Pseudonocardia* sp. genes (4.7% of all analyzed homolog pairs, Supplementary Table 2) that have higher rates of non-synonymous mutations when compared to their homolog in *Pseudonocardia assaccharolytica* (K_a_:K_s_ ≥ 1) because they evolved under a strong divergent selection regime (Figure 4). These genes fall into categories of protein translation (four tRNA methyltransferase modification enzymes and a ribosomal modulation protein), respiration (succinate dehydrogenase), energy storage (acyl CoA dehydrogenase) and membrane transport (polysaccharide, multidrug, potassium, phosphate and cyanate). Other annotations of genes found with a ≥ 1 K_a_:K_s_ratio are more difficult to interpret such as 13 uncharacterized conserved proteins and three transposases, but underscore the potential for discovery of novel microbial traits from understudied environments and taxa. Although this analysis cannot determine the particulars of how these genes differ in terms of the reaction kinetics or substrate specificities of the enzymes they code for, functions like membrane transport and energy storage could plausibly underlie important survival traits for conditions in the nutrient limited high-elevation volcanic deposits of this study.

Another interesting aspect of the K_a_:K_s_ ratio analysis is that only 23% of total volcano *Pseudonocardia* sp. protein coding genes could be unambiguously aligned to homologs from *P. asaccharolytica*. The remaining 77% of genes are too divergent to analyze with this method. This limits the power of the analysis somewhat, but highlights the genetic novelty of each of these organisms, and suggests that further genomic and culture work on the *Pseudonocardia* spp. is warranted.

We find the most abundant genome in the community is intermediately sized (4.9 Mb, not including highly repetitive content, Table 2), and codes for diverse metabolic potential. This size is not unexpected though, as work by Konstantinidis and Tiedje (2004) shows evidence that heterogeneous, variable, and low nutrient niches in soils select for larger genomes, which often contain enhanced regulatory and secondary metabolite synthesis pathways. Barberán et al. (2014) recently expanded this concept by showing that, to some extent, genome size is a reflection of the complexity and variability of terrestrial bacterial niches. Thus, even though utilization of low concentration atmospheric substrates may be important traits for the volcano *Pseudonocardia* sp., we did not expect to find signatures of genome streamlining, as have been documented in oceanic bacteria that specialize in low concentration nutrient uptake (Giovannoni et al., 2014). Given the variability of a high mountain top environment (Lynch et al., 2012) that experiences frequent wet-dry and freeze-thaw cycling stresses (Stres et al., 2010), we are not surprised to find significantly higher numbers of genes classified in the regulation and cell signaling categories in the total metagenome (Figure 2), as well as specific examples of transcription and response regulator genes with high copy numbers (Supplementary Table 1), and with high K_a_:K_s_ ratios (Supplementary Table 2) in the genome of the most abundant community member.

## Conclusions

The functional inferences drawn from this culture-independent study can now serve as testable hypotheses for ongoing culture-based experiments. Although a modest collection of bacteria and fungi have been cultured and isolated from these volcano samples using a variety of selection techniques (unpublished), the most abundant lineages observed from culture-independent approaches have thus far resisted isolation. Nevertheless, the results we present here can inform future culture-based physiological analyses by providing information on potential electron donors and growth conditions.

The atmosphere interfaces with diverse terrestrial and aquatic environments, so it is possible that the pathways and signatures of selection we have detected result from activity and replication elsewhere. Selective dispersal and dormancy processes cannot be ruled out either; perhaps we have recovered genomic material from the most well-dispersing or longest surviving spores. Although there is little evidence to suggest that the most abundant organism from the Llullaillaco Volcano study sites is native to another environment, or is an exceptional spore producer, these are possibilities that cannot yet be rejected, especially considering the evidence for wind borne transport of other lower abundance lineages of the community (Supplementary Figure 1).

Overall, our initial analyses of these metagenomes indicates that despite, or perhaps because of, the intense solar radiation this sparsely populated high-elevation microbial community lacks endogenous photosynthesizing primary producers, but possesses the genetic potential for utilization of various low molecular weight atmospheric substrates and CO_2_ fixation. This seems to support our hypothesis that chemoautotrophic, rather than photoautotrophic, microbes may be supplying organic carbon to simple and low-energy flux communities at these sites, but does not allow us to determine the relative roles that heterotrophic or mixotrophic metabolism may play. Bacterial growth on trace gases and aerosols is difficult to study and can likely support only low rates of metabolism. Answering whether or not the intriguing combination of metabolic pathways found in the volcano *Pseudonocardia* sp. genome indicates an actual dependency for growth on one or more atmospheric substrates requires direct physiological experimentation at relevant gas concentrations. These pathways could also be supplemental to more standard heterotrophic metabolism, and may not by themselves support growth and cell division. Future studies of these high-elevation actinobacteria and their relatives (Cockell et al., 2013; Rhodes et al., 2013) should consider the possibility that a mixture of atmospheric, precipitation and soil derived substrates may be required for growth, or that these organisms are but remnants of extinct ecosystems or windblown transients.

### Conflict of interest statement

The authors declare that the research was conducted in the absence of any commercial or financial relationships that could be construed as a potential conflict of interest.
