# Supplementary material for: Metagenomic evidence for metabolism of trace atmospheric gases by high-elevation desert Actinobacteria
Source: Front Microbiol. 2014 Dec 17;5:698. doi: 10.3389/fmicb.2014.00698 (PMC4269115; doi:10.3389/fmicb.2014.00698)
Supplement: Supplementary file 3 [file DataSheet1.PDF]

***Supplementary Material***

# Metagenomic evidence for metabolism of trace atmospheric gases by high-elevation desert Actinobacteria

Ryan C. Lynch[1,*], John L. Darcy[1], Nolan C. Kane[1], Diana R. Nemergut[2,3,4], Steve K. Schmidt[1]

[1]Department of Ecology and Evolutionary Biology, University of Colorado, Boulder, Colorado, USA
[2]Environmental Studies Program, University of Colorado, Boulder, Colorado, USA
[3]Institute of Arctic and Alpine Research, University of Colorado, Boulder, Colorado, USA
[4]Department of Biology, Duke University, Durham, North Carolina 27708

*Correspondence: R. C. Lynch, University of Colorado, Department of Ecology and Evolutionary Biology, Campus Box 334, Boulder, CO 80309-0334, USA. rlynch@colorado.edu

## 1. Supplementary Figures

Supplementary Figure 1. Maximum likelihood phylogeny of Acidimicrobiae $OTU_{1\%}$ lineages. *Aciditerrimonas ferrireducens* (Itoh et al., 2011) was isolated from geothermal volcanic soils and can respire heterotrophically using a limited spectrum of sugars or reduce ferric iron under anaerobic conditions.

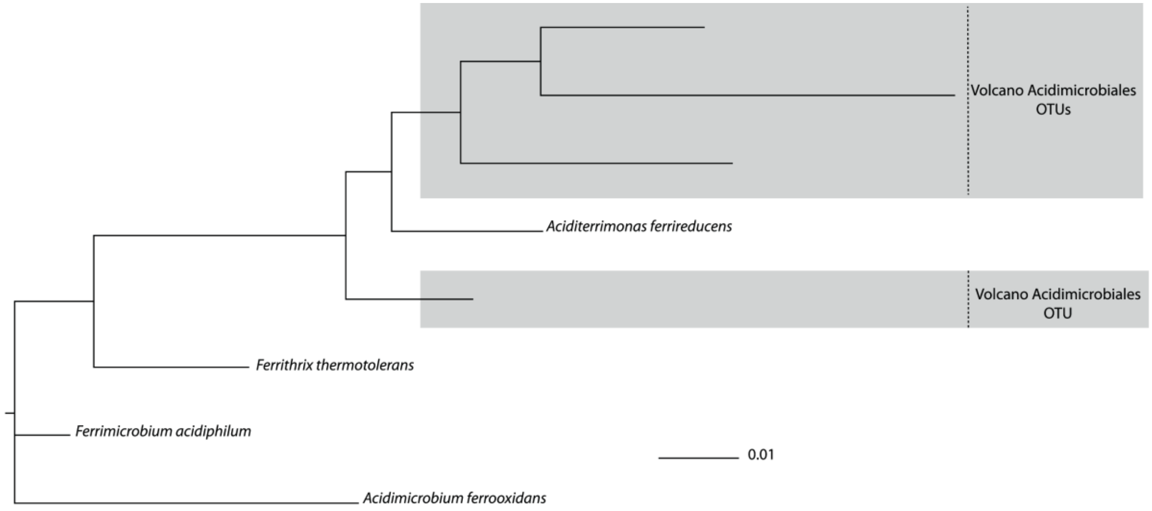

Supplementary Figure 2. K-mer analyses of genomic complexity, sampling effort and identity. A) 15-mer spectrum plot including both Llullaillaco volcano metagenome libraries (yellow and blue) and various publically available reference desert (black) and non-desert (grey) metagenomes. Dataset are publicly available from MG RAST: all datasets from (Fierer et al., 2012) and the Luquillo rainforest metagenome (4446153.3).

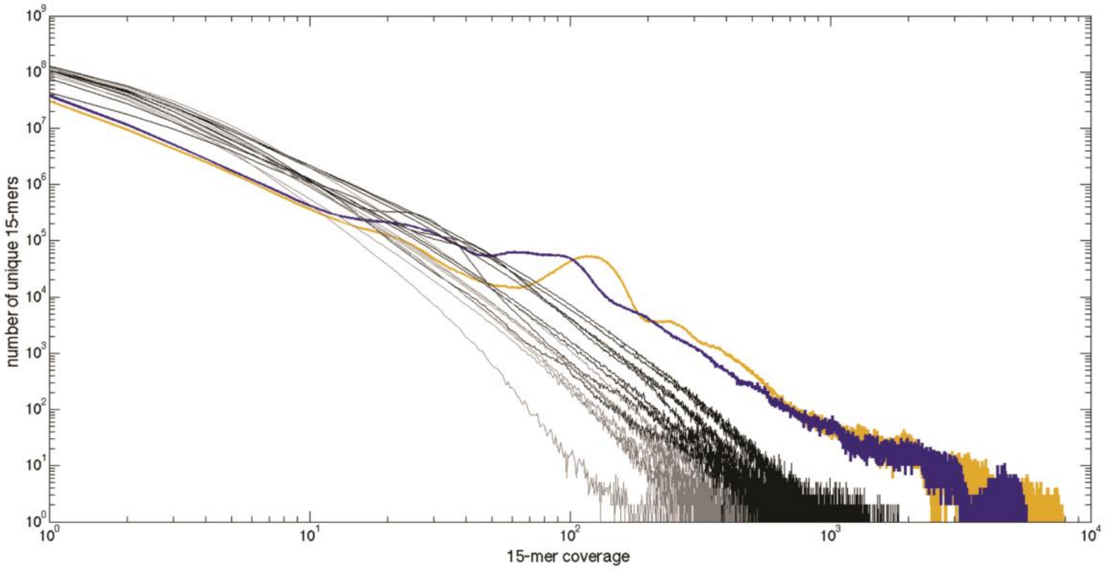

Supplementary Figure 3. Tetramer based emergent self-organizing map, built from the volcano *Pseudonocardia* sp. contigs (large bright green squares), volcano Acidimicrobiae contigs (small green squares) , plus the *Streptococcus coelicolor* genome AL645882, purple squares) as an out group. The topology of this self-organizing map confirms the discriminatory power of the assembly coverage level based bins (Figure S3), particularly for the volcano *Pseudonocardia* sp. The Acidimicrobiae bin contigs appear to contain a higher degree of mixing across topological features, likely representing both misclassifications and transposon driven horizontal gene transfer events.

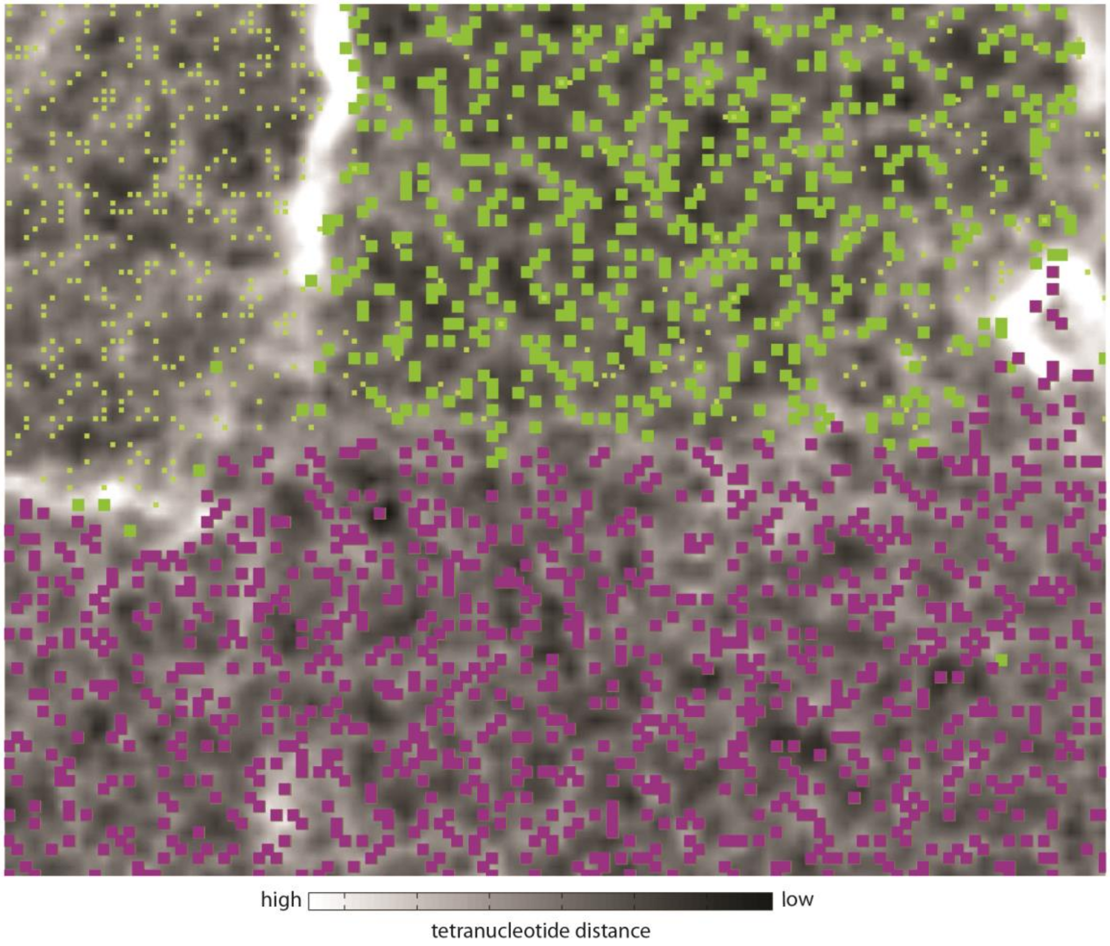

Supplementary Figure 4. Histograms of average coverage levels of contigs from metagenome assembly.

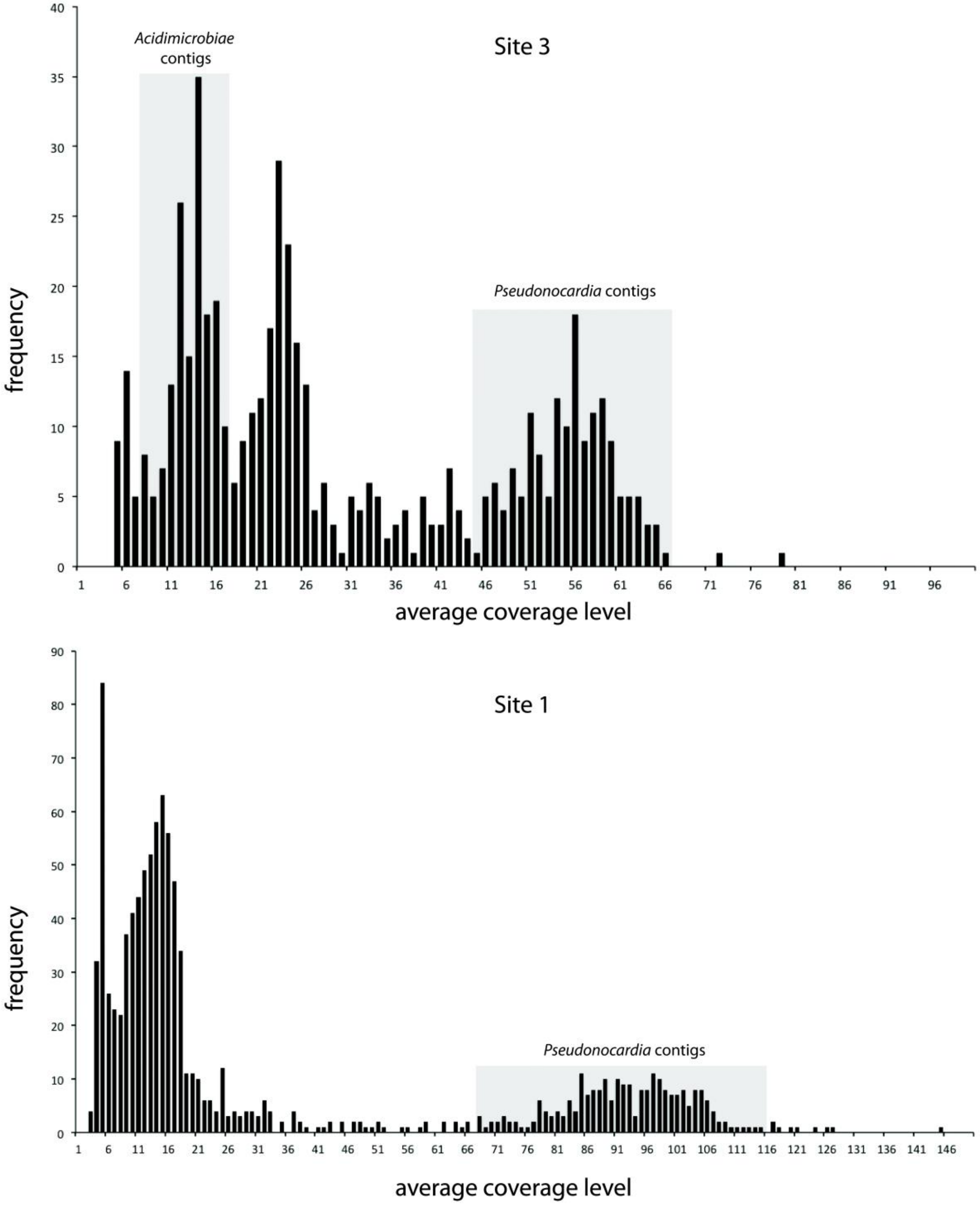

**2. References**

Fierer, N., Leff, J. W., Adams, B. J., Nielsen, U. N., Thomas, S., Lauber, et al. (2012). Cross-biome metagenomic analyses of soil microbial communities and their functional attributes. *Proc. Natl. Acad. Sci. U.S.A* 109, 21390–21395. doi: 10.1073/pnas.1215210110

Itoh, T., Yamanoi, K., Kudo. T., Ohkuma. M., and Takashina. T. (2011). *Aciditerrimonas ferrireducens* gen. nov., sp. nov., an iron-reducing thermoacidophilic actinobacterium isolated from a solfataric field. *Int. J. Syst. Evol. Microbiol.* 61, 1281–1285. doi: 10.1099/ijs.0.023044-0.
